# An asymptomatic flesh-colored tumor on the dorsal hand

**DOI:** 10.1016/j.jdcr.2025.01.030

**Published:** 2025-02-25

**Authors:** Suraj Patel, Milena Dragovic, Ellie Richards, Francisca Kartono

**Affiliations:** a Dermatology, Corewell Health Hospital, Farmington Hills, Michigan; b California Health Sciences University College of Osteopathic Medicine, Clovis, California; c MI Skin Center Dermatology Clinic, Northville, Michigan

**Keywords:** guidelines of management, pleomorphic liposarcoma, soft tissue sarcoma

## Case report

**Fig 1:**
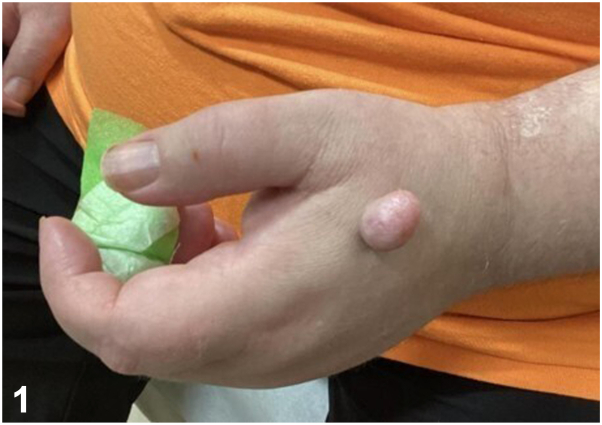

**Fig 2:**
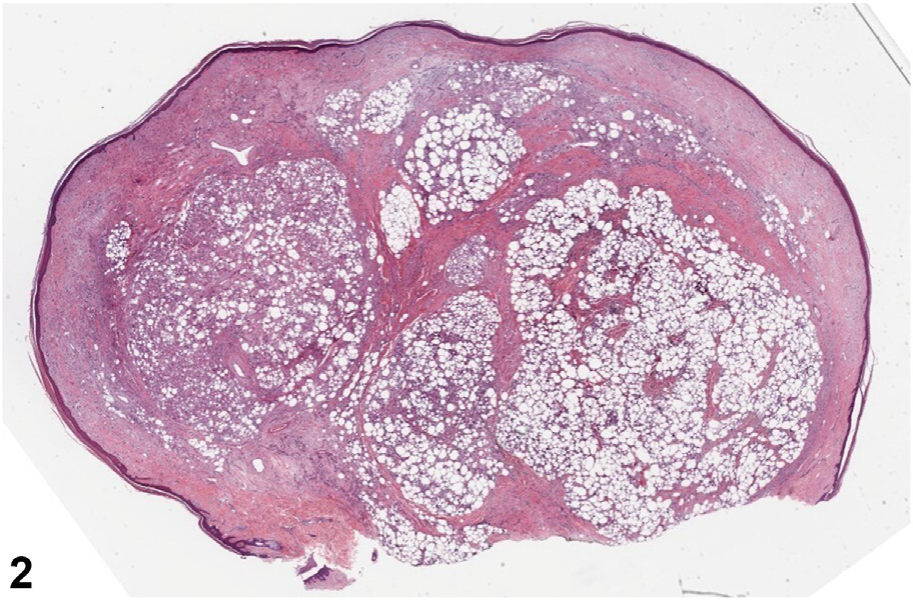

**Fig 3:**
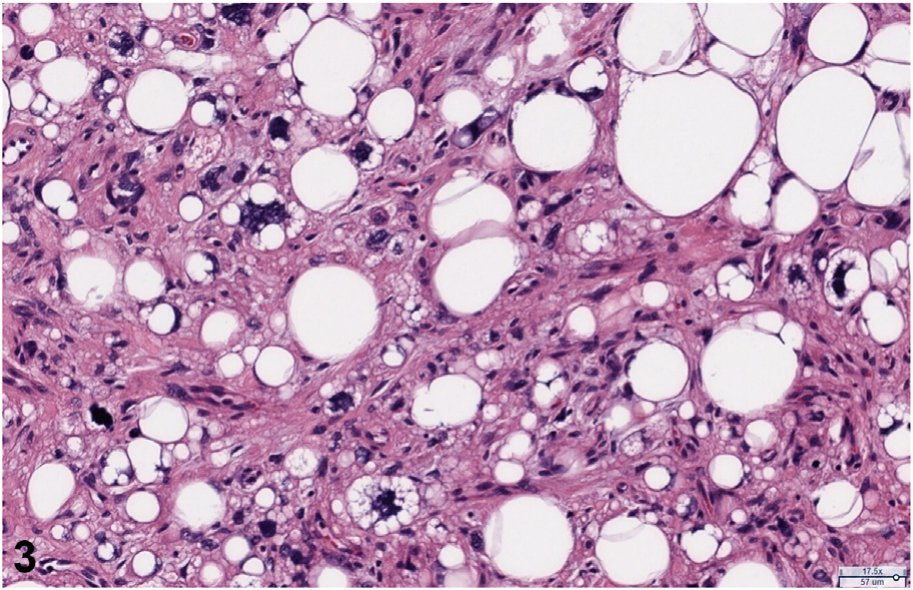

A 61-year-old male with a history of coronary artery disease, osteoarthritis, asthma, schizophrenia, and intellectual disability presented to a dermatology clinic for evaluation of a lesion later diagnosed as a pyogenic granuloma. Further exam revealed a bland 2.5 cm flesh-colored tumor on the dorsal left hand, present for approximately 10 years (Fig 1). A shave biopsy demonstrated a polypoid portion of skin with dermal proliferation of atypical spindled to bizarre pleomorphic cells extending into thickened subcutaneous septae (Fig 2). Higher power magnification revealed subcutaneous lobules containing large pleomorphic lipoblasts with atypical mitotic figures (Fig 3).

**Question 1: Based on the histologic images, what is the most likely diagnosis?**

A. Well-differentiated liposarcoma
B. Pleomorphic fibroma
C. Spindle cell lipoma
D. Pleomorphic liposarcoma
E. Myxoid liposarcoma

**Answers:**

A. Well-differentiated liposarcoma – Incorrect. The biopsy above demonstrates atypical lipoblasts. Histopathology of well-differentiated liposarcoma would reveal mature adipocytes.^1^
B. Pleomorphic fibroma – Incorrect. A pleomorphic fibroma would have a sclerotic background.^2^
C. Spindle cell lipoma – Incorrect. This entity would have mature adipocytes, ropey collagen bundles and bland spindle cells.^3^
D. Pleomorphic liposarcoma – Correct. This is the rarest liposarcoma variant and is diagnosed based on the presence of large pleomorphic lipoblasts with atypical mitotic figures organized in subcutaneous nodules.^1^
E. Myxoid liposarcoma – Incorrect. This entity would demonstrate lipogenic areas, loose basophilic/myxoid stroma, with or without atypical lipoblasts.^1^

**Question 2: What is the best next step in management for patients diagnosed with this condition?**

A. X-ray of involved site to evaluate for bony involvement
B. Evaluation of metastasis with positron emission tomography (PET)-computed tomography CT imaging
C. Chemotherapy
D. Wide-local excision
E. No additional treatment is indicated

**Answers:**

A. X-ray of involved site to evaluate for bony involvement – Incorrect. An x-ray of the site would not be the best next step in management as it would not provide information regarding any systemic spread of this condition.
B. Evaluation of metastasis with PET-CT imaging – Correct. A PET-CT is indicated to further diagnose and stage possible metastasis.^1^
C. Chemotherapy – Incorrect. Chemotherapy would not be the best next step in management because we are unsure if systemic treatment is indicated without first evaluating the presence of local or systemic metastases.
D. Wide-local excision – Incorrect. While a wide-local excision would be indicated to ensure the removal of the primary tumor, further imaging studies must first be conducted to evaluate for metastatic disease.
E. No additional treatment is indicated – Incorrect. Diagnosis of this rare tumor must include evaluation for metastatic disease and long-term surveillance of recurrence.

**Question 3: What immunohistochemical stain profile would help differentiate this entity from other variants?**

A. S100+, MDM2−, CDK4−, DDIT3−, CD34+, p16+, Mutant p53 hyperexpression
B. S100+, MDM2+, CDK+, DDIT3−, CD34−, p16+, p53 wildtype
C. S100+, MDM2+, CDK4+, DDIT3−, CD34−, p16+, p53 wildtype or mutant
D. S100+, MDM2−, CDK4−, DDIT3+, CD 34−, p16−, p53 wildtype
E. S100−, MDM2−, CDK4−, DDIT3−, CD34+, p16+, Mutant p53 hyperexpression

**Answers:**

A. S100+, MDM2−, CDK4−, DDIT3−, CD34+, p16+, Mutant p53 hyperexpression – Correct. This is the expression profile for a pleomorphic liposarcoma.^1^
B. S100+, MDM2+, CDK+, DDIT3−, CD34−, p16+, p53 wildtype – Incorrect. This is the expression profile for a well-differentiated pleomorphic liposarcoma.^1^
C. S100+, MDM2+, CDK4+, DDIT3−, CD34−, p16+, p53 wildtype or mutant – Incorrect. This is the expression profile for a dedifferentiated liposarcoma.^1^
D. S100+, MDM2−, CDK4−, DDIT3+, CD 34−, p16−, p53 wildtype – Incorrect. This is the expression profile for a myxoid liposarcoma.^1^
E. S100−, MDM2−, CDK4−, DDIT3−, CD34+, p16+, Mutant p53 hyperexpression – Incorrect. The S100 negativity makes this answer choice incorrect.^1^

## Conflicts of interest

None disclosed.
